# Effectiveness Analysis of the Non-Standard Reinforcement of Lattice Tower Legs Using the Component-Based Finite Element Method

**DOI:** 10.3390/ma18061242

**Published:** 2025-03-11

**Authors:** Jacek Szafran, Klaudia Juszczyk-Andraszyk, Paulina Kaszubska

**Affiliations:** 1 Department of Structural Mechanics, Faculty of Civil Engineering, Architecture and Environmental Engineering, Lodz University of Technology, Al. Politechniki 6, 90-924 Łódź, Poland; 2 Compact-Project.pl Company, Al. Politechniki 22/24, 90-924 Łódź, Poland; kjuszczyk-andraszyk@compact-project.pl; 3 Faculty of Civil Engineering, Architecture and Environmental Engineering, Lodz University of Technology, Al. Politechniki 6, 90-924 Łódź, Poland; 237032@edu.p.lodz.pl

**Keywords:** FEM, lattice tower, L-sections, reinforcement, telecommunication

## Abstract

This paper presents an analysis of the effectiveness of the existing reinforcement of steel lattice tower legs made of L-sections by expanding to closely spaced built-up members. Due to the significant differences between the standard assumptions and the existing reinforcement, numerical analyses based on the component-based finite element method (CBFEM) were used to estimate the capacity of the existing structure’s tower legs. Geometrically and materially nonlinear stress analysis and linear buckling analysis were performed. The obtained results (stress distribution maps, buckling forms, and corresponding critical forces) were used to modify the geometric parameters of the section of the analyzed tower legs in order to adapt the standard formulas in the calculation procedure. In the analyzed case, distance of the connections between the branches exceeded that indicated in EN 1993-1-1:2005 for the condition concerning the possibility of ignoring the deformation susceptibility in the calculation process. However, it did not result in the separate operation of each branch of the section. Thus, in the case of the analyzed reinforcement, it is possible to neglect the form susceptibility when calculating the buckling resistance of the element. The buckling capacity of the reinforced legs of the tower and the compression capacity of the section of the analyzed structure were calculated according to the method that took into account the results of the numerical analyses. These values are about 35–48% and 30–39% higher, respectively, than the capacity of the unreinforced angle calculated according to EN 1993-1-1:2005 and EN 1993-1-8:2006 standards. Thus, it may be possible to avoid costly and labor-intensive retrofitting of the existing reinforcement to meet the standard requirements. A key issue, and one that is particularly important in light of the lack of standard guidelines aimed at designing reinforcements for telecommunications structures, seems to be the performance of full-scale experimental tests.

## 1. Introduction

Continuous and dynamic development in the field of telecommunications is a matter of course today. Efficient and reliable communications systems are extremely important, especially in the face of the challenges we have been facing recently: the coronavirus pandemic, the war in Ukraine, or natural disasters such as widespread fires and floods.

In order for a telecommunications network to operate efficiently, it is necessary to properly maintain technical facilities, an essential part of which are steel lattice tower structures. Most of the structures of this type located in Poland were erected decades ago when other design standards (Polish Building Standards) were in force. Very often, the structural elements of towers were designed and constructed from L-sections and channels. This was due to the availability and price of these sections (the technology of making tubular elements became widespread a little later) and the relatively easy construction of connections. With the development of technology, more and more specialized equipment is being mounted on towers—radio and spreader antennas, radio modules with cabling, and other equipment necessary for the proper operation of the base station. This has led to an increase in the loads acting on the structures. When these loads are considered in conjunction with changes in design standards, it is often the case that the load-bearing capacity of these towers is insufficient to ensure their continued reliable and safe operation as support structures for radio communication installations. In such a situation, these towers require the construction of reinforcement of individual structural elements, in particular tower legs and bracing members. It is imperative to understand the reinforcement’s operational mechanisms and to accurately assess its effectiveness during the design phase. It is a key factor ensuring the reliability of the telecommunications infrastructure and, consequently, the uninterrupted operation of the network.

### 1.1. Background for the Research

Currently, the use of modern materials, in particular carbon fiber-reinforced polymer CFRP [1], glass fiber-reinforced polymer GFRP [2], or polyurea [3], is attracting increasing scientific interest for the reinforcement of building structures, especially those made of reinforced concrete or masonry. However, as pointed out in [1], the use of this type of materials to reinforce steel structures requires an analysis of many complex factors affecting the effectiveness of these solutions. Given the lack of comprehensive studies and the considerable cost of such reinforcement, it is not a popular solution in engineering practice for steel telecommunications towers.

Typically, the strengthening of lattice tower elements is achieved by expanding the bar cross-section, changing the static scheme (shortening the buckling length, adding diaphragms), and replacing individual elements [4]. It is particularly common for L-sections to be expanded to closely spaced built-up members [5,6,7,8,9,10]. The effectiveness of such a solution depends, for example, on the shape and size of the reinforcing element or how the branches are connected to each other. Therefore, such reinforcements are constantly being tested in various configurations.

Li J. et al. in [11] proposed and analyzed a method of reinforcing a steel angle using a reinforcement plate placed on the inner side of the weak axis of the angle connected by double bolted clamps. The reinforcement effect observed was significant with the capacity increment within 39–174%. However, the reinforcement plate did not participate in the distribution of vertical load and only increased the stiffness of the angle. Only slight effect of the clamp types and clamp distance on bearing capacity was noted. Conversely, the clamp-on installation of an additional reinforcing L-section (using steel clamps) was analyzed in [5], obtaining efficiencies of only 0.5–14.5%, depending on the cross-section of the reinforcing angle and the length of the member tested experimentally. Tan X. et al. in [6] analyzed the effectiveness of reinforcing existing L-section by adding another L-section and connecting them using crossed steel plates connected by bolts. The limit load increased by 26–38% depending on the number of steel plates. In [7], it is pointed out that, when reinforcing an L-section with a second L-section, proper spacing of the battens is crucial. Also, it was indicated that the effectiveness of the reinforcement for connecting L-sections with bolts is questionable, while, according to [8], retrofitting with another L-section connected using cruciform bolted connections provides a high load-sharing effectiveness and increases the stiffness of the element. Nevertheless, Lu C. [8] suggests that larger sections of the reinforcing element, as well as stiffer connections between the branches may lead to increased bearing capacity of the member. In [9], attention is drawn to the problem of modeling bolt slip in the case of a bolted connection between branches. According to the model developed and validated in [10], the size and number of filler plates only affect the flexural buckling of the member, which, in most cases, determines the load-bearing capacity of legs.

Another important aspect in determining the effectiveness of the reinforcement appears to be the impact of preload. Over the years, studies on the effect of loading on a compression member during its reinforcement have been conducted by various researchers, but their results are not conclusive. L. Tall [12] believes that the load-bearing capacity of reinforced elements under load is identical to that of unloaded reinforced elements. Contrary to those results, the analyses of M.Vild and M. Bajer [13,14,15,16,17] and Sizhe W. et al. [18,19], indicate that the load-bearing capacity of a reinforced element under load may be slightly lower or higher than that of its unloaded counterpart. It depends on, among other things, the magnitude of the load and the manner of loss of stability.

Additionally, Vu Q.-Y. et al., in [20] after conducting nonlinear pushover analyses for six different reinforcement scenarios, point out that it is crucial to reinforce all tower legs due to the possibility of wind direction changes.

### 1.2. Research Significance

As indicated above, the various ways in which existing steel structures can be strengthened are a frequently discussed topic by numerous researchers. Unfortunately, the conclusions and recommendations from the research carried out so far are not consistent. Also, existing European standards describe procedures for determining the load capacity of such elements applicable only in cases where a series of assumptions are met. In practice, due to technological constraints, such as the considerable height of the installation work and the impossibility of completely relieving the reinforced elements, they are not always met. The proposed calculation procedures do not consider the situation in which we are dealing with the strengthening of an element, but only one in which the entire closely spaced built-up member is designed from scratch, and the force is applied uniformly to all its branches. As highlighted in [21], a reinforced member often cannot be treated in the calculation in the same way as a member designed from the beginning as reinforced. However, there are currently no standard European guidelines for the calculation of reinforced steel members. In such circumstances, the evaluation of effectiveness depends mainly on the individual analyses conducted by the designer.

The purpose of this article is to discuss one unusual case of a tower leg reinforcement solution that neither fits directly into the guidelines described in the standards nor has an equivalent in the research carried out to date. The authors have taken up the challenge of analyzing the effectiveness of this reinforcement depending on how the branches are connected and verifying the applicability of the standard regulations. Complex numerical analyses were carried out for five models. The forms of buckling, the values of elastic critical forces, and the values and distribution of equivalent stresses in the individual elements of the members were compared. Eventually, a method for determining the load capacity of reinforced elements based on the numerical analyses carried out was proposed. The results may become the basis for further experimental work and the development of a new method of reinforcing structural elements made of L-section bars.

## 2. The Reinforced Structure of a Telecommunication Lattice Tower

**Figure 1:**
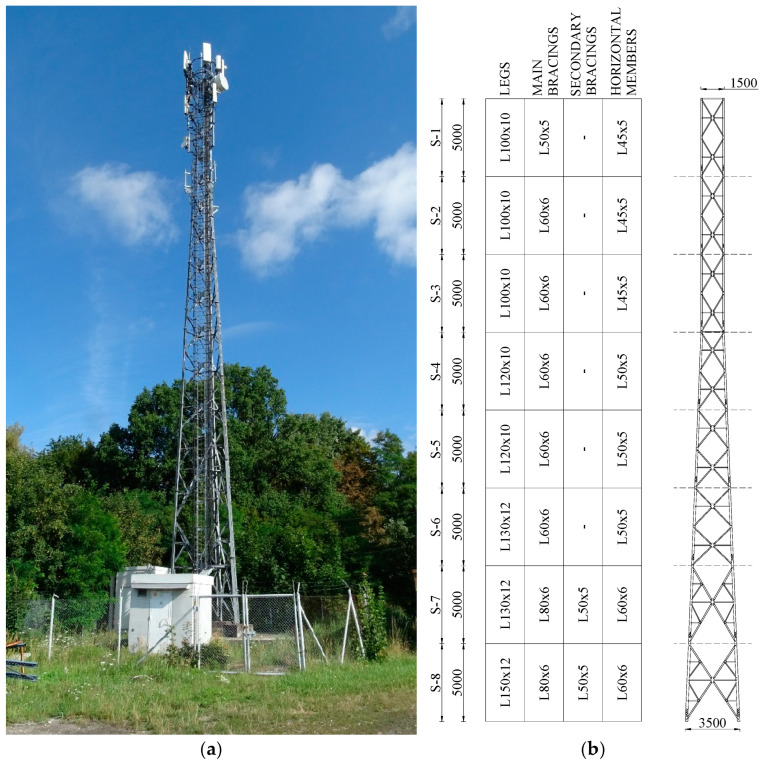
The analyzed structure: (**a**) view of the tower; (**b**) a schematic of the structure.

The telecommunications tower that is the subject of analysis is a steel structure that takes the form of a spatial truss with an equilateral triangle cross-section, whose main structural elements are made of L-section bars (Figure 1). The object was designed and built more than twenty years ago when much less equipment was installed on this type of structure than today. The design was developed in accordance with Polish Building Standards that were in effect prior to the implementation of European standards. Due to the rapid technological advances observed in recent years, constant modernization of the configuration of teletechnical equipment is required. In the case under review, this resulted in a several-fold increase in the windward area of antennas, modules, brackets and cabling. Consequently, there was an increase in internal forces in individual tower elements to values greater than those for which the structure was originally designed. This, in conjunction with the transition from Polish to European standards, resulted in the tower legs not meeting the ultimate load-bearing condition. In order to enable the loads to be carried safely after the subsequent expansion was carried out, it was, therefore, necessary to reinforce these structural elements, which was accomplished.

The main purpose of this article is to evaluate the effectiveness of the existing reinforcement, realized based on the designer’s analysis, unknown to the authors.

**Figure 2:**
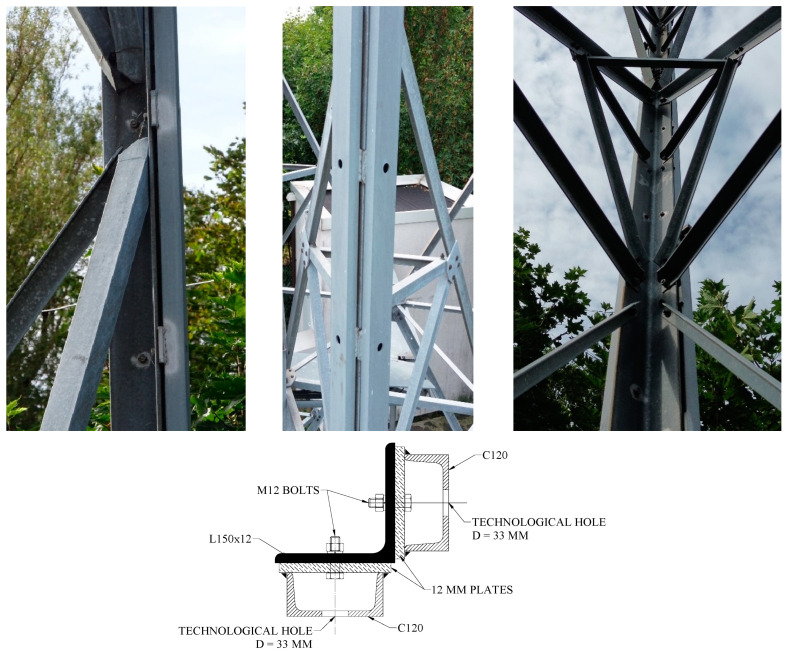
The view of the channel–angle connection (**top**) and cross-section through the reinforced tower leg at the selected location (**bottom**).

Tower legs have been reinforced in a manner that is encountered somewhat less frequently than shortening the buckling length of the strengthened element. This is achieved by expanding the cross-section, a method that is admittedly more labor-intensive and more difficult from the point of view of workmanship. However, this method allows for an increase in the buckling capacity and compression resistance of the section of the strengthened element. The strengthening of the section of the analyzed structure’s tower legs in segments S-3 to S-8 was implemented by mounting two additional branches made of channel sections to the flanges of the existing L-sections (Figure 2), using the sections shown in Table 1. The strengthening branches within a single L-section differ in the location of the joints along the length of the element. There is also a difference in the cross-sections used of the reinforcing elements in the two lower segments along the short sections. The connections of the reinforcing channels to the existing L-sections were made as bolted, using plates welded to the tops of the channel flanges. This solution involved drilling technological holes in the channel sections to allow for the reinforcement to be installed.

**Figure 3:**
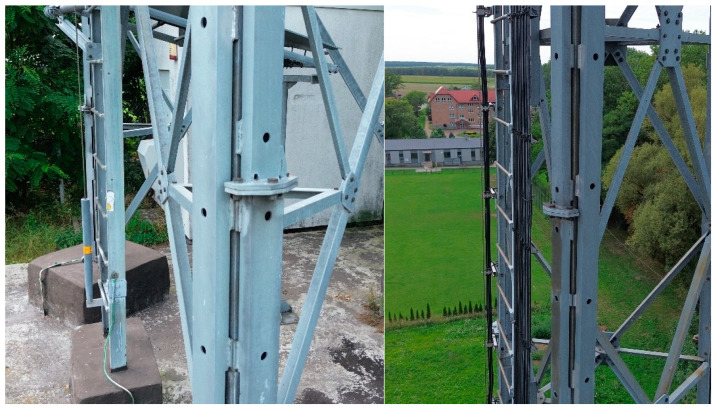
Connections of reinforcing elements along the length.

The connections of the reinforcing channels along their length were designed as bolted butt joints (Figure 3).

**Figure 4:**
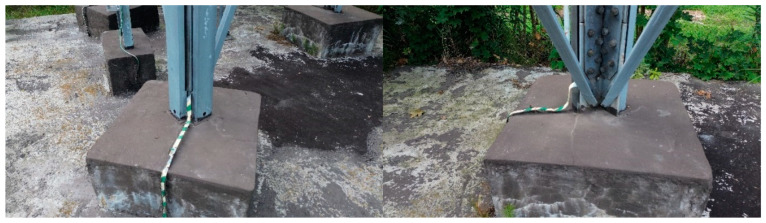
Anchoring of reinforcing elements in the foundation.

The reinforcing elements were anchored to the foundation. For this purpose, the channels reinforcing the tower legs of the tower lowest segment were equipped with “alloy” plates at the base. These plates were designed to transfer the compressive forces occurring in the reinforcing elements directly to the reinforced concrete foundation via a bedding of fine-grained concrete (Figure 4). The detailed parameters of the reinforcement are summarized in Table 2.

## 3. Analysis According to Standards

It is well known that the determination of the load capacity of compression members based on the provisions of European standards consists of calculating the load capacity of the section and analyzing the loss of stability of the element. However, it should be noted that the use of standards formulas and rules requires the fulfillment of a number of assumptions that guarantee the accuracy of the results obtained and the appropriate level of safety of the analyzed structure. Therefore, it is relatively easy and quick to estimate the load capacity of newly designed elements and those made of basic cross sections used in construction. In the case of structures constructed decades ago, which were designed according to standards that have since been withdrawn, and frequently involve individual analyses conducted by the designer or reinforcements of building structures, the design process entails the consideration of numerous performance, technological, and economic factors. Consequently, the calculation of load capacity becomes significantly more complicated. In order to determine its optimal, as close as possible to the actual, and at the same time safe value, some kind of adaptation of the standard provisions based on the experience of the designer, the principles of the art of construction and experimental or numerical analyses is necessary.

### 3.1. Composite Sections Described in European Standards

**Figure 5:**
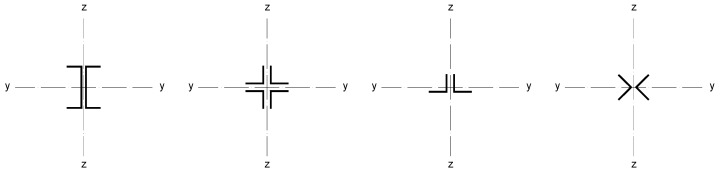
Proximity elements according to the standard [22].

Although the strengthening of compression members by expanding the cross-section is widely used in engineering practice, European standards do not provide explicit guidelines on how to determine the load capacity of such members. The standard [22] only describes methods for composite sections with several identical branches, connected to each other in a strictly defined way. The branches of composite columns should be connected by means of lattice or battens, while closely spaced built-up members (Figure 5) should be in contact or connected through packing plates or pairs of battens in two perpendicular planes. An additional condition also applies to the maximum spacing of connections between the branches of the closely spaced built-up members, the fulfillment of which allows the formability to be ignored when calculating the buckling resistance of the element. This spacing is 15 i_min_ for bolted or welded connections and 70 i_min_ for battens, respectively, where i_min_ is the minimum radius of inertia of one branch. It should be noted that these cases described in the standard apply to newly designed elements, while the strengthening of existing structures involves a number of additional restrictions. First of all, as a rule, the installation of reinforcement is carried out under the operating conditions of the structure, so it is impossible to fully relieve the load on the reinforced elements. In addition, in the case of high structures, which telecommunications towers undoubtedly are, there are a number of technological limitations related to the difficulty of performing certain activities. Among other things, welding, ensuring the permanent connection of elements, and the proper transfer of forces between them is problematic at a considerable height.

### 3.2. Analysis of Standard Conditions

The reinforcement of the analyzed structure is atypical both in terms of the cross-sections of the reinforcing elements used and the way they are connected to the reinforced L-sections. Comparing the realized reinforcement with the standard requirements cited above, a number of discrepancies can be noted. The first and primary one is that the additional branches were made from a different cross-section than the reinforced element. Further differences are related, among other things, to the way the channels are connected to the L-section, which has not been implemented via pacing plates or cross-arranged battens. Also, the spacing of the existing bolted connections is greater than the maximum spacing that allows for ignoring the form susceptibility, as determined by the standard [22] (in places even twice) (Table 3).

Another doubt about the applicability of the standard rules in the case under review is caused by the fact that these rules apply to newly designed elements, that is, when the load is applied from the beginning to all branches of the closely spaced built-up member. Reinforcements of telecommunications towers are performed during their operation when these towers perform their function as support structures for telecommunications equipment. This means that at the time of installation of additional branches in the L-sections, there was a certain state of stress caused, among other things, by the effects of wind on the structure and equipment or by the dead weight of the installed equipment. It is clear that structural strengthening should not be carried out in adverse weather conditions. The current regulations prohibit installation work at wind speeds above 10 m/s in order to ensure the safety of the installers, as well as to minimize the impact of external loads on the structure. However, due to the duration of assembly work and the possibility of unpredictable weather changes, it is not possible to completely eliminate climatic impacts. Given that the channels were installed concurrently with the operation of the structure, it is imperative that the transmission of forces from the L-sections to the reinforcing elements must be executed exclusively through joints between the branches. Therefore, it can be concluded that the manner in which the connection between the reinforcing element and the reinforced element is shaped, as well as its stiffness and load-bearing capacity, are the key parameters that determine the interaction of the channels with the L-section. Consequently, these parameters influence the load-bearing capacity of the tower leg after reinforcement. Determining the proportion of the load that is distributed among the additional branches requires an understanding of the stress distribution in the L-section and channels, which can only be obtained through experimental studies. One possible approach to approximate the stress state is to map actual solutions in numerical analysis.

The analyzed case of reinforcement does not meet the standard assumptions both in terms of the sections used and the way the reinforcing and reinforced elements are connected and the way the load was applied (the force was not applied to all branches at the same time). In situations where there are significant differences between the current state and standard assumptions, using the standard formulas may lead to inaccurate results. Due to the spacing between the bolted connections of the channels with the L-section, the analyzed reinforcement would have to be considered as not meeting the standard requirements allowing for the calculation of buckling resistance with the omission of the form susceptibility. The standard [22] fails to specify the radius of inertia to be employed in the analysis of the loss of stability of the element, as well as the methodology for conducting subsequent calculations.

## 4. CBFEM Numerical Analysis

In order to understand the near-real mechanism of operation of the existing reinforcement and to verify whether it is possible to adopt increased load-bearing capacity of the elements while maintaining an adequate level of the structure’s safety, a series of numerical analyses were carried out. An attempt was made to determine the level of cooperation between branches and to identify the key factors in determining the effectiveness of reinforcement and the load-bearing capacity of elements after reinforcement.

### 4.1. FEM Shell Models for Buckling and Structural Analysis

The reinforced elements were modeled in the IDEA StatiCa 23.1 Member software, which is used to perform complex stability analyses of individual structural elements, taking into account, among other things, the influence of geometric imperfections, material nonlinearities, or finite rotational stiffness of nodes [23]. The program is based on the component-based finite element method (CBFEM), according to which the analyzed elements were modeled as shells of four-node quadrilateral shell finite elements with nodes at corners and six degrees of freedom at each node—three translations (u_x_, u_y_, u_z)_ and three rotations (φ_(x)_, φ_(y)_, φ_(z)_) [24]. Deformations of the element are divided into the membrane and the flexural components [25], and the formulation of membrane behavior is based on the work of Ibrahimbegovic [26].

The analysis for each of the models created was carried out in three stages. It began with materially nonlinear analysis (MNA), followed by linear buckling analysis (LBA), and concluded with the most complex, geometrically over materially nonlinear analysis (GMNIA). A series of checks were conducted to ascertain the stresses arising in the individual components of the reinforced tower leg, their respective deformations, the form of buckling, and the critical forces. Each analysis was carried out in an iterative manner, gradually increasing the applied load until the full load set in the input parameters of the analysis was reached or until the components exceeded the ultimate limit state, defined by the deformation’s limit, which was assumed equal to 5% as recommended by the standard [27].

#### 4.1.1. Materially Nonlinear Analysis (MNA)

Materially nonlinear and geometrically linear static analysis is characterized by a limited range of applicability, related to the slenderness of the analyzed elements and susceptibility to local and global loss of stability [28,29]. This type of analysis takes into account nonlinearities of the model material but does not consider buckling parameters or geometric imperfections. Its results formed the basis for LBA and GMNIA analyses.

#### 4.1.2. Linear Buckling Analysis (LBA)

Linear buckling analysis was used to determine several most probable forms of buckling based on the Lanczos algorithm [30]. At this stage of the analysis, the structure was considered ideal both in terms of geometry (ignoring geometric imperfections of the system) and material (assuming a perfectly elastic steel model) [31]. The results of this analysis, in the form of deformations of the elements presented on the 3D model, provided important information regarding which part of the model and how it experiences a loss of stability, as well as whether the buckling of the reinforced and the reinforcing branches is dependent on each other. For each of the determined forms of buckling, the coefficient α_cr_ was determined, expressing the ratio of the elastic critical force of the element to the value of the load set in the computational case under study, which made it possible to compare these forces between models.

#### 4.1.3. Geometrically and Materially Nonlinear Analysis with Imperfections (GMNIA)

Geometrically and materially nonlinear analysis with imperfections represents the most complex type of analysis for static loads [32]. In this step, both material and geometrical nonlinearities are taken into account, obtaining the most accurate possible determination of the actual stress distribution in the element and its response to a given load. Using this method allows for a more optimal, yet safer, design of structures [33], and with the development of specialized software, it is becoming increasingly popular [31,34,35,36,37]. In order to obtain accurate results from GMNIA analysis, it is essential to adopt appropriate values, shapes, and directions for equivalent geometric imperfections. These imperfections are substitutes for all imperfections in the considered system, including, but not limited to, initial deformations, residual stresses, assembly inaccuracies, and material inhomogeneities. These imperfections emerge during the prefabrication and assembly of the structure and affect the buckling capacity of the elements [32,38]. Researchers are making many attempts to define explicit guidelines on how to take imperfections into account, allowing for accurate and safe results [38,39]. In this study, the maximum amplitude of equivalent geometric imperfections was determined for selected (most probable) forms of buckling, based on the guidelines of the standard [22]. Local bow imperfections, introduced as input parameters of the analysis, were calculated as

e_0_ = L/200 (1)

where L is the buckling length of the element. As a result, bow imperfections were obtained equal to:

- 25 mm for models that do not include bracing members;
- 6 mm for a model including bracing members.

Each time, the value of the imperfection amplitude was assigned to the first form of buckling, characterized by the smallest critical force coefficient, considering all possible directions of the imperfection.

### 4.2. Computational Models

**Figure 6:**
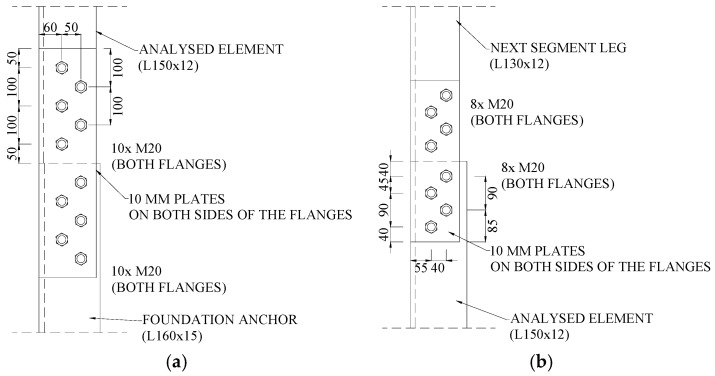
Connections at the ends of the tower leg: (**a**) bottom; (**b**) top.

A total of five models of reinforced tower legs were created. Four models, each measuring 5 m in length, excluding the tower bracings, differed in the manner in which the reinforcing branches were connected to the reinforced L-section, thereby enabling a study of the effect of this connection on their cooperation and the form of buckling. Additionally, a fifth model, measuring 5 m in length, included the tower bracings as so-called related members. This model was designed to be the most accurate representation of the existing tower leg. The connections at both ends of the tower legs were modeled based on the archival design of the analyzed structure, as lap bolted connections with M20 bolts (Figure 6). This took into account the significant effect of their finite rotational stiffness on the buckling length of the elements [40,41,42].

At one end, all degrees of freedom were blocked, while at the other end (to which the compressive force was applied), the ability to move in line with the axis of the bar was provided to allow the load to be transferred to the analyzed tower leg. To each of the models created, a compressive force of 350 kN was applied at the node. The value of the force was chosen so that, for each model, all stages of the analysis were fully carried out (i.e., no plasticization of the element or node plates occurred at less than 100% of the force). This allowed us to compare the obtained results between models. In all models, the tower leg, reinforcement elements (channels), and gusset plates, as well as any associated elements, were modeled as made of S235 steel.

#### 4.2.1. Computational Models That Do Not Include Bracing Members

**Figure 7:**
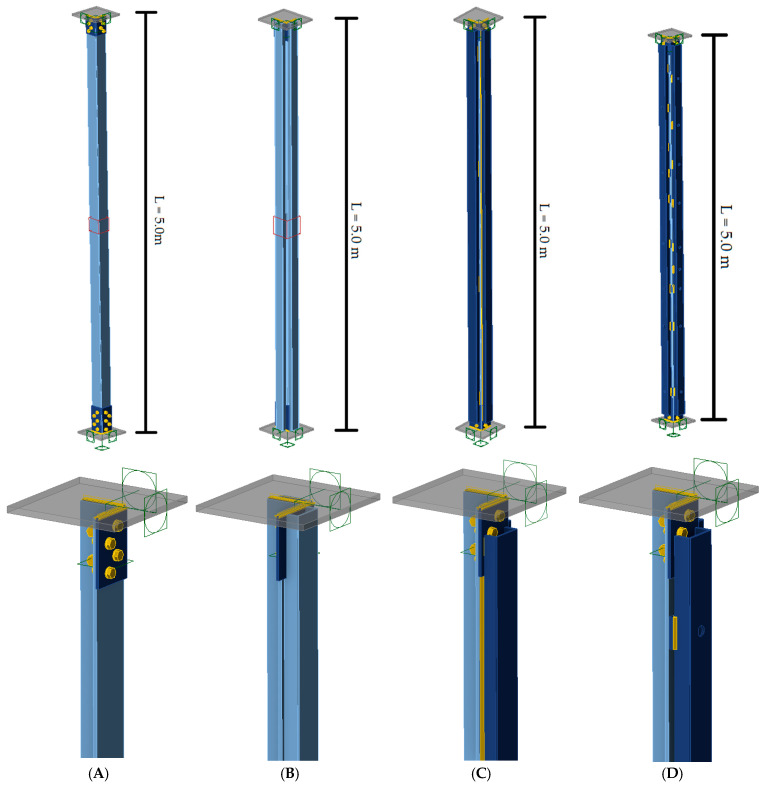
Computational models that do not include elements. (**A**) Model A (**B**) Model B (**C**) Model C (**D**) Model D.

The characteristics of the models not including the bracing members (Figure 7) are shown in Table 4. Model A corresponded to the unreinforced tower leg and served as the reference model for the remaining analyses. In Model B, the reinforced tower leg was modeled as a composite section, ensuring that the L-section and channels fully cooperated and that both the L-section and channels were connected to the theoretical support elements. The consequence of modeling the element in this way was the simultaneous application of compressive force to all its branches. In model C, the channels were modeled as stiffening elements welded to the L-section, while model D represented the actual existing reinforcement, i.e., the channels connected to the L-sections by bolted connections. In models C and D, the reinforcing channels were not connected to the support members, and the compressive force was applied only to the L-section, as is the case in the existing tower leg.

#### 4.2.2. A Computational Model That Takes into Account Bracing Members

**Figure 8:**
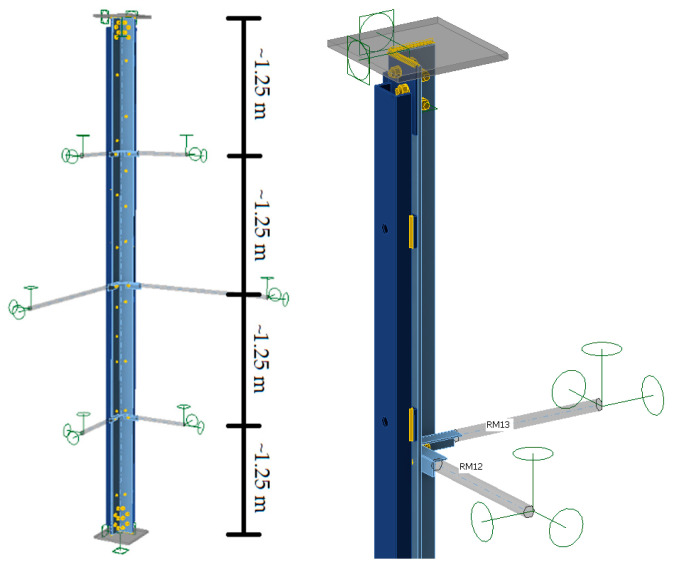
Computational model considering the lattice element.

Model E (Figure 8) took into account the actual tower leg reinforcement—channels connected to the L-section with bolts. Bracings were modeled as related members divided by the Idea Statica Member program into the stub part adjacent to the analyzed member and simplified part at the rest of the related member. The stub was modeled by shell elements (full CBFEM model) and simplified parts by simple 1D beam elements with six degrees of freedom [23]. The bracings at the mid-span of the tower leg were assumed to be made of L60×6 isosceles L-sections, connected to the tower leg with M16 bolts, while the bars at a distance of about 1.25 m from the supports were modeled as L50×5 L-sections connected to the tower leg with M12 bolts. At the ends of the bracing elements, non-sliding pinned support was modeled—i.e., the possibility of sliding along three axes (u_x_, u_y_, u_z)_ was blocked, while the possibility of rotation around these axes (φ_x_, φ_y_, φ_z)_ was released. The channels in the E model were not connected to the support nodes, and the compressive force was applied only to the angle. Detailed characteristics of the model are shown in Table 5.

### 4.3. Mesh

**Figure 9:**
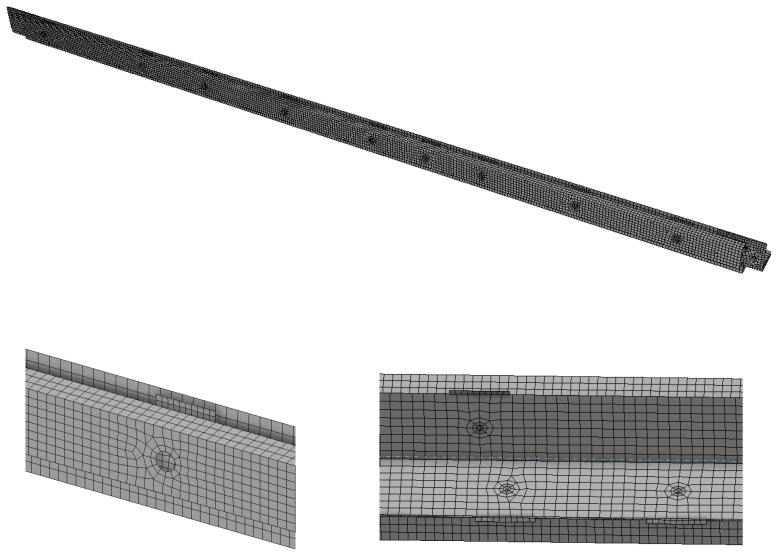
Mesh—general view (**top**), details (**bottom**).

Quadrangular finite elements were used (Figure 9). On the largest web or flange of the element, 8 finite elements were adopted each time. The maximum element size in the models was, therefore, approximately 20 mm. For plates, 10 finite elements were modeled along the shorter side of the plate (element size approx. 12 mm).

### 4.4. Material Model

**Figure 10:**
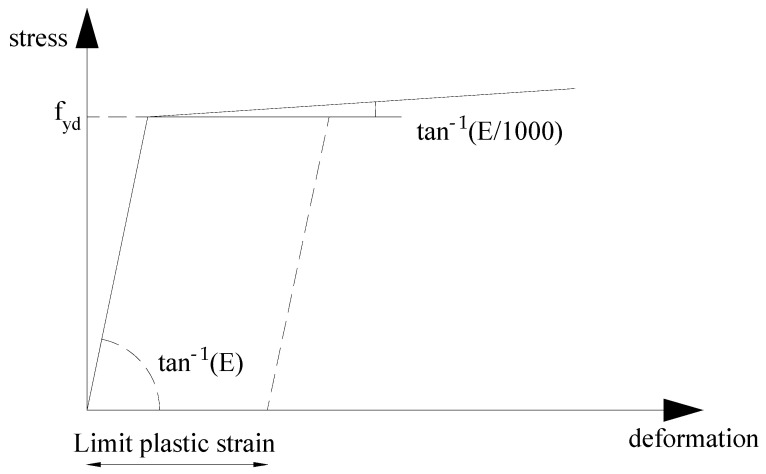
Material model in MNA and GMNIA analyses.

The stress analyses carried out used an elastic–plastic steel model with a nominal slope of the yield plateau according to [27] equal to tan^−1^(E/1000) where E is the elastic modulus of the steel (Figure 10). The behavior of the material was based on the von Mises criterion of plasticity, assuming that it is elastic before reaching the design yield stress f_yd_ [24].

For the linear buckling, perfectly elastic steel model was used.

## 5. Results

As a result of the numerical analyses, equivalent stress maps were obtained, allowing for an approximate assessment of the degree of cooperation between the reinforced L-section and channels. The effect of the method of connecting the branches on the nature of the stress distribution (its inhomogeneity) was observed, as well as on the values of stresses in the channels in relation to the values of stresses in the L-section. The value of maximum stresses for each model was compared, as well as the maximum value of stresses in the L-sections and channels at the mid-length of the analyzed models. For each model, an analysis was conducted to ascertain the proportion of the maximum stress values that occurred in the half-length of the model that was attributed to the L-section, compared to the proportion that was distributed among the individual channels.

Buckling forms and critical force coefficients were obtained as a result of buckling analyses. The first buckling form and the corresponding critical force value for models A–D were compared, paying particular attention to how the critical force changes with an increase in the stiffness of the connections and whether the buckling of the branches of the analyzed elements is mutual.

### 5.1. Stress Analysis (GMNIA)

**Figure 11:**
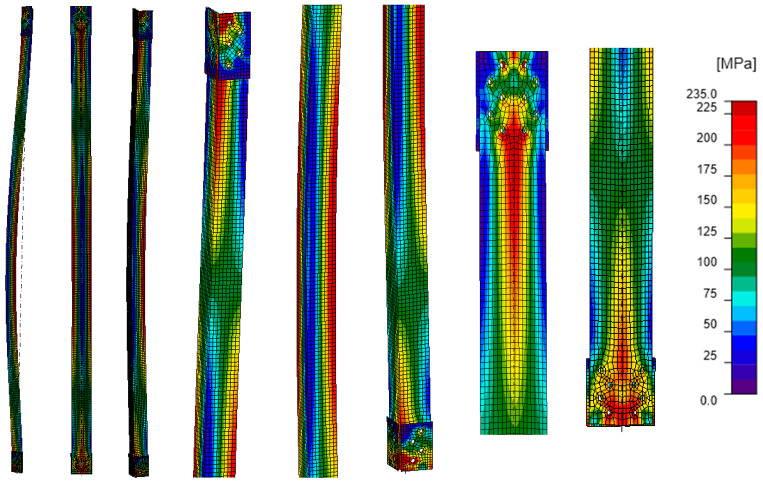
Model A: equivalent stresses; GMNIA analysis—general view (left), close-ups on the element (center), close-ups on connections (right).

**Figure 12:**
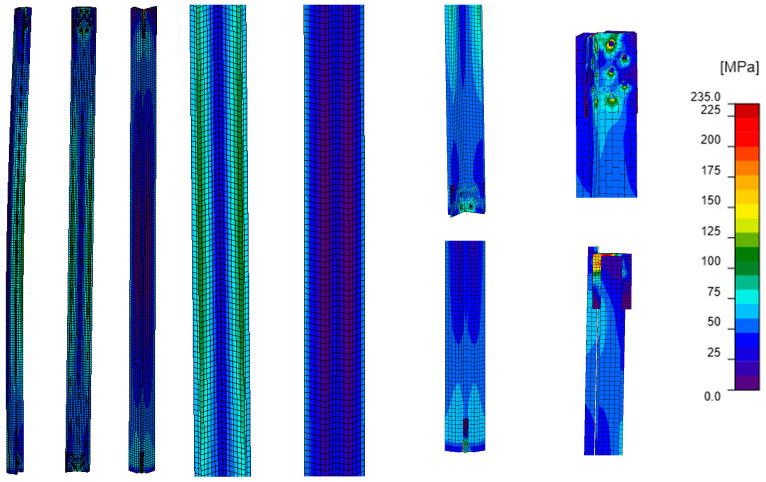
Model B: equivalent stresses; GMNIA analysis—general view (left), close-ups on the element (center), close-ups on connections (right).

**Figure 13:**
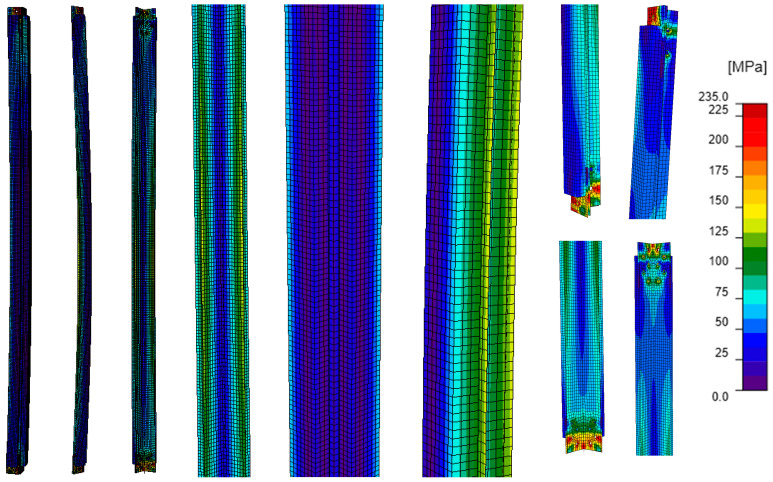
Model C: equivalent stresses; GMNIA analysis—general view (left), close-ups on the element (center), close-ups on connections (right).

**Figure 14:**
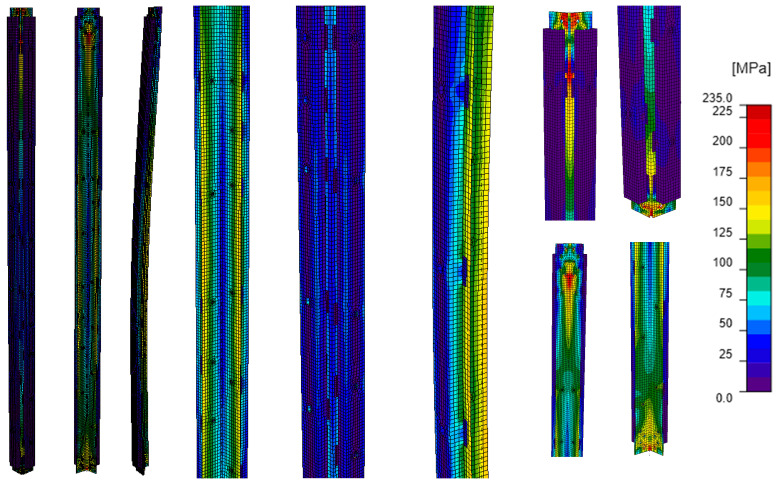
Model D: equivalent stresses; GMNIA analysis—general view (left), close-ups on the element (center), close-ups on connections (right).

Comparing the equivalent stress maps for model A (Figure 11) with those for models B–D (Figure 12, Figure 13 and Figure 14), it can be observed that the addition of branches made of channels to the existing L-section results in a reduction in stresses along the length of the L-section in each of the analyzed cases. For an unreinforced angle bar, the equivalent stresses along the length reach values close to the yield strength of the steel. In the other models, part of the stresses is distributed among the channels. The value of stresses in the reinforcing elements compared to the value of stresses in the L-section depends on the way the branches of the reinforced tower leg are connected (Table 6). The greatest reduction in stress in the L-section occurs in model B, in which the channels form a single section with the L-section, and the load is applied to all its branches. In fact, this case corresponds to an element whose branches are all welded together, and the compressive force is applied to the center of gravity of the section. In contrast, the smallest stress reduction occurs when the branches are connected by bolts (model D, a model that is the closest to the existing reinforcement). Based on the maximum stresses at the half-length of the modeled tower legs, it can be seen that regardless of the way the channels are connected to the L-section, the higher stresses occur in the L-section. They account for 38% to 44% of the sum of the maximum stresses occurring in the L-section and two channels, while the stresses in a single channel are about 28–31% of the sum of the maximum stresses in the analyzed section.

The way the branches are connected also strongly determines the stress distribution in the element. Model B is characterized by the greatest homogeneity of stresses (Figure 12). Almost throughout the whole model, they reach values between 50 and 110 MPa. The exception is the connection of the analyzed element to the support element where a local increase in stress values is clearly visible. In models C and D (Figure 13 and Figure 14), the stress values in the element are more varied. Model C is characterized by an apparent increase in stresses in the reinforcing channels along the entire length of the weld connecting them to the L-section, while in model D, local increases in stresses can be observed in the area of fasteners and process holes.

**Figure 15:**
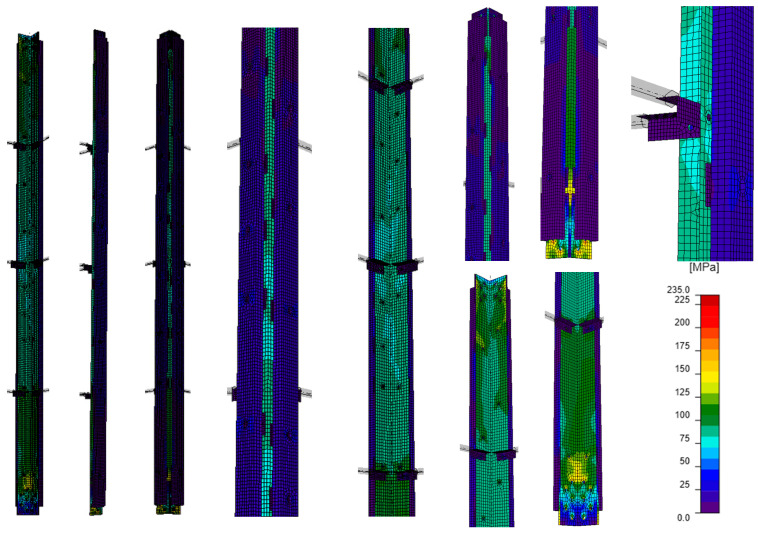
Model E: equivalent stresses; GMNIA analysis—general view (left), close-ups on the element (center), close-ups on connections (right).

The stress distribution obtained by GMNIA analysis for model E (Figure 15) is characterized by inhomogeneities analogous to those obtained for model D (Figure 14). Also, in this case, a local increase in the value of stresses in the channels near the process holes can be observed. However, the difference is the value of stresses themselves, which are smaller in both L-section and channels of model E compared to model D. Analyzing the maximum stress values in the L-section and channels in the selected section located in the middle of the L-section length (Table 7), it can be seen that the stress in the L-section is about 60% of the sum of the maximum stress values, while the stress in a single channel is about 20% of the sum of these stress values. The lower percentage of stresses in the single channel, compared to the models that do include the bracing members, indicates the limited cooperation between the channels and the L-section.

### 5.2. Buckling Analysis (LBA)

Table 8 summarizes the results of the LBA analysis for models A–D (models that do not include bracing members). For all models, the first form of buckling is global flexural–torsional buckling of the element. An important observation, particularly for model D, is that all the branches of the element (L-section and reinforcing channels) deform together as one element. There is no separation of the branches (increase in the distance between the elements of the section) between the nodes connecting them. It is clear that the buckling of the branches in each model is mutual.

Using the critical force coefficients derived for each model from IdeaStatica Member, the elastic critical force was calculated (Table 8), based on a well-known formula:

F_cr_ = α_cr_N (2)

where α_cr_ is the critical force coefficient, and N is the compressive force applied to the node.

The critical force of the unreinforced L-section is significantly smaller compared to the critical forces of models B–D. The largest critical coefficient and the largest elastic critical force are characterized by model B. The values of the critical forces for models C and D are between those obtained for models A and B, with the critical force in the case of channels welded to the L-section (model C) reaching a higher value than in the case of the bolted connection between the channels and the L-section (model D). Thus, the results obtained confirm the relationship between the stiffness and load-bearing capacity of the connection between the branches and the effectiveness of the reinforcement. The welded connection, which is stiffer compared to the bolted connection analyzed, results in a higher elastic critical force.

## 6. Assessment of the Load Capacity of the Analyzed Structure

### 6.1. Compressive and Buckling Resistance According to European Standards

The resistance of individual bracing members of telecommunication towers is calculated based on the provisions of standards [22,43]. The compression resistance of the section is described by the formula:

N_Rd_ = A · f_y_/γ_(M1)_, (3)

while the buckling capacity by the formula:

N_b,Rd_ = χ · A · f_y_/γ_(M1)_ (4)

The parameters summarized in Table 9 were used to determine them for the tower legs of the analyzed structure without considering the existing reinforcement.

The value of relative slenderness λ_rv_, which is strongly correlated with the radius of inertia of the cross-section of the element, is described according to the standard [22] by the following expression:

(5) $$\lambda_{\mathrm{rv}}=\frac{L_{\mathrm{cr}}}{i_{v}}*{\left(93.9*\sqrt{\frac{235}{f_{\mathrm{dy}}}}\right)}^{-1}$$

in which L_cr_ is the buckling length equal in the case of the analyzed structure to 1.25 m, i_v_ is the section’s radius of inertia with respect to the weak axis, and f_dy_ is the dimensionless yield strength of steel equal to 235.

### 6.2. Calculation Procedure Taking into Account the Results of Numerical Analyses

The results of the numerical analysis indicate that there is a certain level of cooperation between the reinforcing channels and the reinforced L-section, which can result in an increased load-bearing capacity of the structure. Its estimation was of the utmost importance in determining the safety of increasing the surface area of the teletechnical equipment mounted on the tower.

It was decided that the results of the numerical analyses would be incorporated into the process of determining the geometric parameters of the cross-section of the analyzed elements. This would include the cross-sectional area and the radius of inertia, which have been shown to significantly influence the load-bearing capacity of the element. A larger cross-sectional area is associated with the ability of the cross-section and the element to transfer greater compressive forces. Furthermore, the reduction in slenderness has a beneficial effect on the buckling capacity of the element, reducing its susceptibility to loss of stability. Thus, in the calculation procedure for reinforced tower legs, it was possible to adapt Formulas (3) and (4) and the parameters shown in Table 9.

Given that the compressive load is applied directly only to the L-section within the actual structure, it was determined that in the modified calculation procedure, the cross-sectional area of the entire L-section and part of the cross-sectional area of each channel should be taken in each case. This approach accounts for the incomplete cooperation between the branches resulting from the method used to connect them. In order to estimate the cooperating area of the channels, the relations between the stresses in the L-section and channels, obtained from the GMNIA analysis for the model E, presented in Table 6, were used. Based on them, it was calculated that the stresses in the L-section are almost three times greater than those in the single channel and account for 60% of the sum of the maximum stresses in the selected section, while the stresses in the single channel are 20% of this sum. Since the tower leg models in the numerical analysis are subjected only to a compressive force, the stress value is directly proportional to this force and inversely proportional to the cross-sectional area and is expressed by the well-known formula:

σ_xc_ = N_(c)_/A_(xc)_ (6)

where σ_xc_ is the normal compressive stress, N_c_ is the compressive force, and A_xc_ is the area of the cross-section under compression. The cross-sectional area of an L150×12 L-section is comparable to the cross-sectional area of two C120 channels. Given this, under certain simplifications that disregard inequalities in the distribution of stresses, it can be assumed that the force distributed among each channel is three times lower than the force taken by the L-section. Formulas (3) and (4) are true under the assumption of uniform stress distribution throughout the cross-section A. In order for this assumption to be fulfilled, it was decided that the mating cross-sectional area would include the L-section area and one-third of the area of each channel.

The results of the buckling analyses indicated that the L-section and channel sections of model D deform together despite the non-standard joint spacing. In view of this, in the computational procedure developed, it was decided to disregard the form susceptibility of the elements and treat the L-section and two channels as a uniform section under buckling. This effect was obtained by taking as the radius of inertia i_v_ the radius of inertia of the entire closely spaced built-up section with respect to its weak axis. The geometric parameters of the selected section determined based on the new calculation procedure that incorporated the results of the numerical analyses are shown in Table 10.

### 6.3. Bearing Capacity Comparison

Table 11 summarizes the load capacities of the tower legs determined for an unreinforced L-section, i.e., assuming as the cross-sectional area A the area of the L-section itself and as the radius of inertia i_v_ the radius of inertia of the L-section alone with respect to its weak axis, and using modified geometric parameters according to the calculation procedure developed from the results of the numerical analyses.

Buckling capacities determined based on the modified calculation procedure are greater by about 37–48% compared to those of an unreinforced L-section, while section capacities are greater by about 30–39% (Table 12).

## 7. Discussion

- Based on the GMNIA, all reinforced models analyzed are characterized by a lower stress value along the length of the L-section between nodes compared to the unreinforced L-section. Nevertheless, as the stiffness of the connection increases, the stress values decrease. For example, in model C, stress value in the L-section along its length is about 23% lower than in model D.
- The way the branches are connected strongly determines the stress distribution in the element. For example, in model C, an apparent increase in stresses in the reinforcing channels along the entire length of the weld connecting them to the L-section occurred. Whereas in model D, local increases in stresses can be observed in the area of fasteners and process holes.
- Based on the LBA, the analyzed closely spaced built-up member is characterized by a higher elastic critical force compared to the unreinforced one regardless of the method of connection between the branches. However, the elastic critical force of such an element is greater the greater the stiffness of the connection between the branches of the section is. The difference in critical force for a welded channel–angle joint (model C) versus a bolted one (model D) is about 11%.
- For the analyzed case, the use of the spacing of joints between branches greater than that indicated in the standard [22] for the condition concerning the possibility of ignoring the form susceptibility in the calculation course did not result in the separate operation of each branch of the section. Therefore, in the case of the analyzed reinforcement, it is possible to ignore the susceptibility when calculating the buckling resistance of the element at a distance between joints greater than the maximum spacing determined by the standard [22].
- The buckling capacity of the reinforced tower legs of the analyzed structure calculated according to the procedure, taking into account the results of numerical analyses, is higher by about 35–48% compared to the capacity of the unreinforced L-section calculated on the basis of standards [22,43], while the compression capacity of the section is higher by about 30–39%.

## 8. Conclusions

This paper presents an analysis of the effectiveness of the existing reinforcement of steel lattice tower legs made of L-sections by expanding to a closely spaced built-up member. Due to the significant differences between the standard assumptions and the existing reinforcement, CBFEM-based numerical analyses were used to estimate the capacity of the existing structure’s tower legs. Geometrically and materially nonlinear stress analysis and linear buckling analysis were performed. The obtained results (stress maps, buckling forms and corresponding critical forces) were used to modify the geometric parameters of the section of the analyzed tower legs in order to adapt the standard formulas in the calculation procedure.

The simulations and calculations carried out in this work formed the basis for the following conclusions:

- The effectiveness of reinforcement involving the expansion of tower legs to a closely spaced built-up member depends largely on how the reinforcing elements are connected to the reinforced one (particularly on the stiffness and load-bearing capacity of the connection); as the stiffness of the connection increases, the elastic critical force of the element increases and the stress value in the reinforced L-section decreases.
- The European standards’ guidelines for closely-spaced built-up members cover only strictly defined cases and involve numerous assumptions, making their applicability to unusual reinforcement solutions rather limited.
- Complex reinforcement analysis supported by a proper validation process can result in the possibility of avoiding costly and labor-intensive retrofitting of the existing reinforcement to meet the requirements of the standards.

Modern design support tools can be extremely useful in evaluating existing structural reinforcements, as evidenced by the results of the analyses presented in this paper. Due to the technical limitations of the software regarding the non-inclusion of connection load capacity in the element stability analysis, the results obtained can be used for a preliminary assessment of the element’s behavior under compressive loading and comparison between the models. In order to determine the load capacity of the analyzed element, it would be additionally necessary to carry out a detailed analysis of the load capacity of the connections between the branches of the element and at its ends, considering, among other things, the effect of contact stresses and frictional forces. Another important issue is to verify the accuracy of the models and assumptions made in the analyses described in the paper by conducting appropriate real-scale experimental tests. The performance of full-scale experimental studies, therefore, appears to be a key issue and particularly important in light of the lack of standard guidelines aimed at the design of reinforcements for telecommunications structures.

## Figures and Tables

**Table 1:** Cross-sections of tower legs and reinforcing elements.

Tower Segment	Section of Reinforced Angle Bar	Cross-Section of Reinforcing Channels
S-3	L100×10	2×C80
		2×C100
S-4	L120×10	2×C100
S-5	L120×10	2×C100
S-6	L130×12	2×C100
S-7	L130×12	2×C100
		C100 + C1200
		2×C120
S-8	L150×12	2×C120
		C120 + C140
		2×C140

**Table 2:** Parameters of the realized tower leg reinforcement.

Combination of channel bars with L-sections	Plate thickness	12 mm
	Diameter of the hole in the axis of the plate	13 mm
	Diameter of the hole in the channel	33 mm
	Joint spacing	from 0.286 m to 0.83 m
	Bolts	M12
Joints of channel sections along the length	Plate thickness	14 mm
	Bolts	3×M20

**Table 3:** Characteristics of the analyzed near-branch section.

Cross-Section	i_min_	15 i_min_
	[cm]	[cm]
L150×12	2.95	44.25
C120	1.59	23.85

**Table 4:** Characteristics of the analyzed computational models that do not include bracing members.

Model Symbol	A	B	C	D
Cross-sections of the analyzed elements	L150×12	L150×12 + 2×C120	L150×12 + 2×C120	L150×12 + 2×C120
Basic parameters of the model	-	-	welds: 6 mm	welds: 6 mm
				screws: M12 cl. 5.8
				max. screw spacing: 0.85 m
				sheet metal: 140 × 120 × 12
Cross-section through the model	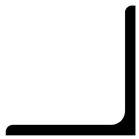	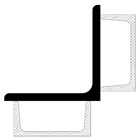	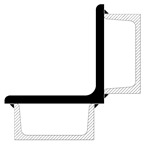	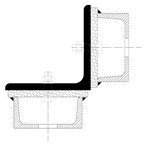

**Table 5:** Characteristics of the analyzed computational model considering bracing members.

Model Symbol	E	
Cross-sections of the analyzed elements	L150×12 + 2×C120	
Basic parameters of the model	welds	6 mm
	sheet	140 × 100 × 12 mm
	screws	M12 cl.5.8
	max. bolt spacing	0.85 m
	trussing	L60×6/L50×6
Cross-section through the model	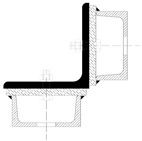	

**Table 6:** Stress values in the elements for models A–D.

	Model A	Model B	Model C	Model D
Maximum stresses [MPa].	235.2	178.0	235.0	212.7
Maximum stresses in the L-section in the selected section [MPa].	235.0	110.0	135.0	175.0
Maximum stresses in a single channel in the selected section [MPa].	-	80.0	110.0	110.0

**Table 7:** Stress values in the elements for the model E.

	Model E
Maximum stresses [MPa].	172.7
Maximum stresses in the L-section in the selected section [MPa].	115.0
Maximum stresses in channels in the selected section [MPa].	40.0

**Table 8:** Buckling analysis results for models A–D.

	Model A	Model B	Model C	Model D
First form of buckling 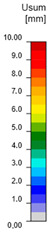	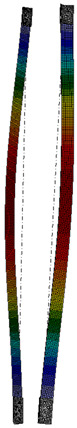	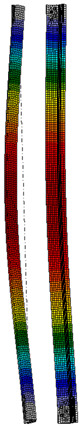	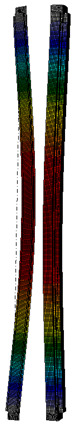	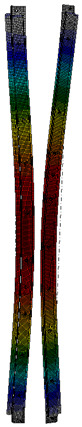
Critical force coefficient α_cr_ [-].	2.16	4.47	4.17	3.75
Critical force F_cr_ [kN].	756.0	1564.5	1456.5	1312.5

**Table 9:** The basic parameters used in calculating the compression resistance of the section and the buckling capacity of the element according to European standards.

Parameter	Notation	Value
A	cross-sectional area	-
f_y_	yield strength of steel	235 MPa
γ_M1_	safety factor	1.0
χ	buckling ratio	$1*{\left(\Phi +\sqrt{\Phi^{2}-{\lambda^{2}}_{eff}}\right)}^{-1}$
ϕ	rate	$0.5*[1+\alpha *\left(\lambda_{eff}-0.2\right)+{\lambda^{2}}_{eff}]$
α	imperfection parameter of the buckling curve b	0.34
λ_eff_	effective slenderness	$k*\lambda_{rv}$
k	effective slenderness ratio	$\max\{0.8+\frac{\lambda_{rv}}{10};\;0.9\}$

**Table 10:** Geometric parameters of the section in the computational approach based on the results of numerical analyses.

Components of a Compound Section	L150×12 + 2×C120
L-section area [cm^2^].	34.8
Cross-sectional area of one channel [cm^2^].	17.0
Cross-sectional area of one mating channel	5.7
Total area included in the load capacity calculation [cm^2^].	46.2
Radius of inertia of the composite section with respect to the weak axis [cm].	3.82

**Table 11:** Summary of the load capacity of the tower legs determined for the unreinforced angle and according to the computational approach based on the results of numerical analysis.

Cross-Sections of Elements		Load Capacities [kN]			
		The Normative Approach—A Non-Enhanced Element		An Approach Based on the Results of Numerical Analysis	
L-Section	Channels	Buckling	Section	Buckling	Section
L100×10	2×C80	374.39	451.20	555.46	621.81
L120×10	2×C100	480.22	545.20	698.99	754.59
L130×12	2×C100	632.07	705.00	854.28	914.39
L130×12	C100 + C120	632.07	705.00	887.83	941.53
L130×12	2×C120	632.07	705.00	919.37	968.67
L150×12	2×C120	755.35	817.80	1037.22	1081.47
L150×12	C120 + C140	755.35	817.80	1065.78	1107.84
L150×12	2×C140	755.35	817.80	1094.31	1134.20

**Table 12:** Summary of the increase in the load capacity of the tower legs compared to the unreinforced element.

Cross-Sections of Elements		Bearing Capacity Increment [%]	
L-Section	Channels	Buckling	Section
L100×10	2×C80	48.4	37.8
L120×10	2×C100	45.6	38.4
L130×12	2×C100	35.2	29.7
L130×12	C100 + C120	40.5	33.6
L130×12	2×C120	45.5	37.4
L150×12	2×C120	37.3	32.2
L150×12	C120 + C140	41.1	35.5
L150×12	2×C140	44.9	38.7

## Data Availability

The original contributions presented in this study are included in the article. Further inquiries can be directed to the corresponding author.
